# Selective Expansion of Skeletal Muscle Stem Cells from Bulk Muscle Cells in Soft Three‐Dimensional Fibrin Gel

**DOI:** 10.1002/sctm.16-0427

**Published:** 2017-02-28

**Authors:** Pei Zhu, Yalu Zhou, Furen Wu, Yuanfan Hong, Xin Wang, Gajendra Shekhawat, Jeffrey Mosenson, Wen‐Shu Wu

**Affiliations:** ^1^ Division of Hematology/Oncology, Department of Medicine and Cancer CenterUniversity of Illinois at ChicagoChicagoIllinoisUSA; ^2^ Department of Materials Science and EngineeringNorthwestern UniversityEvanstonIllinoisUSA

**Keywords:** Myogenic progenitor cells, Muscle stem cells, Three‐dimensional fibrin gel, Satellite cells

## Abstract

Muscle stem cells (MuSCs) exhibit robust myogenic potential in vivo, thus providing a promising curative treatment for muscle disorders. Ex vivo expansion of adult MuSCs is highly desired to achieve a therapeutic cell dose because of their scarcity in limited muscle biopsies. Sorting of pure MuSCs is generally required for all the current culture systems. Here we developed a soft three‐dimensional (3D) salmon fibrin gel culture system that can selectively expand mouse MuSCs from bulk skeletal muscle preparations without cell sorting and faithfully maintain their regenerative capacity in culture. Our study established a novel platform for convenient ex vivo expansion of MuSCs, thus greatly advancing stem cell‐based therapies for various muscle disorders. Stem Cells Translational Medicine *2017;6:1412–1423*

**Significance Statement**

Muscle stem cells (MuSCs) expansion is essential for stem cell‐based therapies to treat muscle disorders. Here, we established a soft three‐dimensional fibrin gel culture system for selective expansion of MuSCs from bulk muscle cells without the need for cell sorting, highlighting the potential of this culture system as a platform for advancing the intervention of effective MuSC‐based muscular genetic disease therapies via CRISPR/Cas9‐based genome editing of MuSCs.

## Introduction

Skeletal muscle is a form of striated muscle tissue involved in diverse bodily functions. The functional unit of skeletal muscle is the multinucleated myofibers formed by the fusion of hundreds of myoblasts in a process called myogenesis. During postnatal myogenesis, satellite cells are responsible for growth and regeneration of muscle fibers 1. Satellite cells are morphologically identified as mononucleated cells lying between basal lamina and myofiber plasma membrane. Because satellite cells can self‐renew 2 and differentiate into functional progeny 3, 4, there is a consensus that they are bona fide adult muscle stem cells (MuSCs).

In healthy unstressed muscles, most MuSCs are mitotically quiescent while transcriptionally active 5, 6, 7. Quiescent MuSCs are characterized by their expression of Pax7 and/or Myf5 but not MyoD and myogenin (MyoG) 7. Upon muscle injury or detachment from basal lamina, MuSCs become activated and initiate rapid expression of myogenic transcription factor MyoD, which enables cells to re‐enter the cell cycle and proliferate 8, 9, 10. Proliferating satellite cells and their progeny are often referred to as myogenic precursor cells. The ratio of Pax7 to MyoD in satellite cells is important in determining the fate of activated satellite cells (ASC). An intermediate ratio of Pax7 to MyoD keeps satellite cells proliferating without differentiation, while a low Pax7‐to‐MyoD ratio drives stem cells into terminal differentiation 7.

Transplantation‐based studies have demonstrated the impressive regenerative potential of MuSCs in repairing damaged or diseased muscle 11, 12, 13, 14, bringing to light the importance of these adult stem cells as therapeutic targets in treating inherited, acquired, and age‐associated muscular disorders. Highly enriched mouse MuSCs can now be prospectively obtained by fluorescence‐activated cell sorting (FACS) or magnetic‐activated cell sorting (MACS) using unique combinations of various cell surface markers of CD45^−^Sca‐1^−^Mac‐1^−^CXCR4^+^integrin‐β1^+^ 11, 15, CD45^−^Sca‐1^−^CD11b^‐^CD31^−^CD34^+^integrin‐α7^+^ 13 or CD31^−^CD45^‐^Sca‐1^−^VCAM^+^ 16. However, the scarcity of native adult MuSCs residing in muscle tissues makes impractical their direct therapeutic application. Therefore, ex vivo expansion of regenerative myogenic cells is highly desired.

Unfortunately, isolated satellite cells either lose their regenerative potential 13 or have a limited capability to proliferate 17 upon culture on traditional Matrigel‐coated substrate. The suspension/spheroid culture was reported to propagate muscle‐derived stem cells in a form of myosphere 18, 19. However, compared to satellite cells, myosphere‐derived cells were deemed more “pre‐myogenic” in nature, with transdifferentiation potentials not limited to muscle cells 20. There were several other attempts for improving either regenerative capacity or self‐renewal of ex vivo expanded MuSCs. For instance, hydrogels mimicking native muscle elasticity increased proliferative ability of isolated MuSCs 21. Signaling molecules such as Wnt7a 22 and Notch 23 were shown to affect satellite cell behavior and fate by driving the symmetric expansion and promoting self‐renewal, respectively. Forskolin‐induced elevation of cyclic AMP 24 as well as inhibition of JAK‐STAT activity 25, 26 or p38 mitogen‐activated protein kinase 27, 28 markedly stimulated proliferation of cultured satellite cells and enhanced their engraftment potentials after transplantation. In addition, a cocktail of four pro‐inflammatory cytokines secreted by T cells was reported to support long‐term in vitro culture of functional MuSCs 29. Regardless of physical or biochemical strategies for expanding adult MuSCs described above, these studies purified MuSCs by either FACS or MACS, which requires a relatively large number of cells and relies on troublesome antibody staining and washing steps. These sorting techniques increase the risks for contaminations and potentially alter cell properties like gene expression and regenerative capacity 30, thus hampering the progress of MuSC‐based therapies. Therefore, an innovative culture system enabling selective propagation of MuSCs from a small number of bulk skeletal muscle cells and faithful maintenance of their regenerative capability will undoubtedly be crucial for the development of stem cell‐based therapies.

In this study, we developed a soft three‐dimensional (3D) salmon fibrin gel culture system by reaction of fibrinogen and thrombin, and showed that this system selectively expanded adult mouse MuSCs from bulk skeletal muscle preparations without the need for prior cell sorting. We demonstrated that this culture system exclusively expands MuSCs and maintains their regenerative properties. Therefore, this novel 3D fibrin gel culture system provides a platform to advance stem cell‐based therapies for the treatment of muscle disorders.

## Materials and Methods

### Mice

C57BL/6, NOD/SCID immunodeficient, C57BL/6‐Tg(CAG‐EGFP)10sb/J, *Pax7^tm1(cre)Mrc^*/J, *ROSA^mT/mG^*, and C57BL/10ScSn‐*Dmd^md^x*/J mice were obtained from Jackson Laboratories. All experiments were performed with 4‐ to 8‐week‐old mice, and in compliance with the institutional guidelines of University of Illinois at Chicago.

### Mouse Skeletal Muscle Cell Preparation

Hindlimb tibialis anterior (TA) and gastrocnemius muscles were dissected, cleaned by removal of non‐muscle tissues including tendons, fat, and vessels, followed by cutting into small fragments and being subjected to collagenase (100 U/ml, Catalog no. 171001015, Life technologies, Carlsbad,) and dispase (2 U/ml, Catalog no. 17105041, Life technologies, Carlsbad) digestions in DMEM at 37°C water bath for 1 hour. After enzymatic digestion, muscle mass was resuspended in DMEM with 10% horse serum, and further mechanically triturated through vigorous and consecutive passing through a 10‐ml glass pipette and subsequently 9′′ cotton‐plugged glass Pasteur pipette. Resultant cell suspension was passed through sequential 70‐μm and 40‐μm strainer (BD Biosciences, San Jose) to generate bulk skeletal muscle single‐cell suspensions.

### Mouse Satellite Cell Sorting

To divide different populations in mouse skeletal muscle cells, FACS was performed as described by Sacco et al. 13. Briefly, the mononucleated cells were firstly stained with biotinylated antibodies reactive to CD45, CD11b, CD31, and Sca1. Next, cells were incubated cells with streptavidin‐APC‐Cy7, integrin‐α7 antibody labeled with PE (MBL, Woburn and eFluor660‐conjugated CD34 antibody (eBioscience, San Diego). Cells negative for CD45, CD31, CD11b, Sca1, and positive for CD34 and/or integrin‐α7 were sorted as MuSCs, while those negative for the lineage markers as well as CD34 and integrin‐α7 were used as non‐MuSCs.

### Skeletal Muscle Cell Culture

Mouse skeletal muscle cells were cultured using Dulbecco's Modified Eagle Medium (DMEM)/F10 (1:1) with 20% Fetal bovine serum (FBS), 2.5 ng/ml basic fibroblast growth factor (bFGF) (PROSPEC, East Brunswick) and 1% penicillin/streptomycin. For coating 24‐well plate with Matrigel, proper volume of stock Matrigel (BD Biosciences, San Jose) was diluted into 1 mg/ml working solution by ice‐cold DMEM in a prechilled 50‐ml conical tube on ice. Diluted Matrigel solution (150 μl) was then added into 24‐well plate through a chilled 1‐ml glass pipette. Coated‐plates were left on ice for 7 minutes followed by incubation in tissue culture incubator for at least 1 hour after removal of working Matrigel solution in each well. For preparing 3D fibrin gel culture of muscle cells, salmon fibrinogen (Sea Run Holdings, Freeport) was diluted into gradient concentrations. For one well of 24‐well plate, 125 μl fibrinogen and 125 μl cell solution were mixed together. A total volume of 250 μl cell/fibrinogen mixture was transferred into each well of 24‐well plate and mixed well with preadded 5 μl salmon thrombin (Sea Run Holdings, Freeport) (0.1 U/μl). The plate was then moved into 37°C cell culture incubator for no less than 30 minutes to allow sufficient gel forming reaction. Afterward, 1 ml growth medium were added, and replenished every other day. To induce differentiation, cells were switched from growth medium into fusion medium (DMEM supplemented with 2% horse serum) for 3 days.

### Quantification of Cell Growth Rate and Differentiation

Bulk skeletal muscle cells or sorted satellite cells were seeded in Matrigel‐coated 24‐well plate or mixed with fibrinogen and plated in thrombin preadded 24‐well plate and cultured in growth medium. After 7 days of culture, cells were trypsinized and counted using a hemocytometer. Cell growth rate was displayed in the form of fold increase in cell number. For cells expanded from sorted satellite cells, fold increase in cell number was calculated by dividing total progeny number with the initial seeding number, while for cells expanded from bulk muscle cells, initial seeded MuSCs number in bulk cells was evaluated by FACS first, by which, total progeny number was divided to get the fold change of cell number. For quantitation of differentiation, myoblasts/myotubes were costained with anti‐myosin antibody and 4′,6‐diamidino‐2‐phenylindole (DAPI), and the fusion index was determined as an average percentage of nuclei within myosin positive cells versus total number of nuclei from three randomly captured fields. Nuclei number was counted by ImageJ plugin software.

### Transplantation of Muscle Cells

Host NOD/SCID mice were anesthetized by intraperitoneal injection of Ketamine (100 mg/kg body weight) and Xylazine (10 mg/kg body weight). For muscle injury and transplantation, recipient mice were anesthetized with isoflurane (Merial, Lyon, France). TA muscles were injected with 50 μl cardiotoxin (*Naja mossambica mossambica*, 10 μM; Sigma, St. Louis) 1 day before transplantation. Next day, fresh satellite cells, bulk cells or gel‐expanded progeny were resuspended in 15 μl of PBS and then injected into preinjured TA muscle. When indicated, an equal number of freshly sorted satellite cells and bulk skeletal muscle cells from GFP‐transgenic mice were injected as controls. Muscles were harvested from recipient mice 4 weeks after transplantation and analyzed by cryo‐sectioning and microscopy. To quantify GFP‐positive fibers, Continuous sectioning on the whole TA muscle transplanted with donor‐derived cells were checked using epifluorescence for GFP. Cryo‐sections with most intense GFP epifluorescence sigal were subject to immunohistochemistry using specific antibodies against laminin (indicating sarcolemma boarders) and GFP (indicating donor cell‐derived myofibers) for counting the number of GFP‐positive fibers.

### Microarray Analysis

Total RNAs extracted using Quick RNA MicroPrep kit (ZYMO Research, Irvine) were first analyzed by Bioanalyzer (Agilent Technologies, Santa, Clara). A triplicate of RNA samples with an RNA integrity number > 9.0 were used for subsequent labeling and hybridization with Mouse Gene 2.0 ST Arrays (Affymetrix, Santa Clara). Expression data was processed using Gene Expression Consol (Affymetrix, Santa Clara) or standard methods in R. The source code used the *heatmap* and *heatmap.2* command within the *gplots* package of the R programming language to create the heatmap. Principal component analysis (PCA) was performed using NIA Array Analysis tool (http://lgsun.grc.nia.nih.gov/ANOVA/index.html) 31, 32 to generate 2‐dimensional biplots. Gene ontology analysis was conducted in The Database for Annotation, Visualization and Integrated Discovery v6.7 (DAVID, http://david.abcc.ncifcrf.gov) using all detectable genes as the background. Only enriched pathways with FDR value < 0.05 were presented.

### Data Analysis

All the experimental data are presented as mean ± SEM. Significant differences between means for single comparisons were determined by unpaired two‐tailed student's *t* test.

## Results

### Soft 3D Salmon Fibrin Gel Favorably Supports Growth of Skeletal MuSCs

MuSCs offer the potential to treat muscular disorders including muscle atrophy, therefore, it is highly desired to expand these cells from limited biopsy sources in vitro. A two‐dimensional (2D) hydrogel culture system was previously shown to expand freshly sorted MuSCs without losing regenerative capacity for 7 days 21. Due to the 3D microarchitecture niche of MuSCs in the body, we hypothesized that a 3D culture system would facilitate ex vivo expansion of MuSCs. After considering the features of several gel matrixes such as thermoresponsive Mebiol gel 33, synthetic nanofibrillar cellulose hydrogel 34, and injectable RGD‐alginate gel 35, we selected salmon fibrin gel for our initial studies because it can be easily prepared from polymerization of native fibrinogen and thrombin, and is adjustable in elasticity and rigidity 36. In addition, salmon fibrin gel is nontoxic and has low immunogenicity, therefore is highly applicable for use in medicine and bioengineering 37, 38. When using fibrin gel to culture the early passage primary skeletal muscle cells that consist mainly of myoblasts and fibroblasts, we found that a low concentration (0.5 mg/ml) of 3D salmon fibrin gel favored growth of round myoblasts even in fibroblast‐favorable DMEM/F10 containing growth medium (Supporting Information Fig. S1). By contrast, higher concentrations of fibrin gel supported the proliferation of fibroblasts. Here, we designate 0.5 mg/ml of 3D salmon fibrin gel as “soft” 3D fibrin gel.

**Figure 1:**
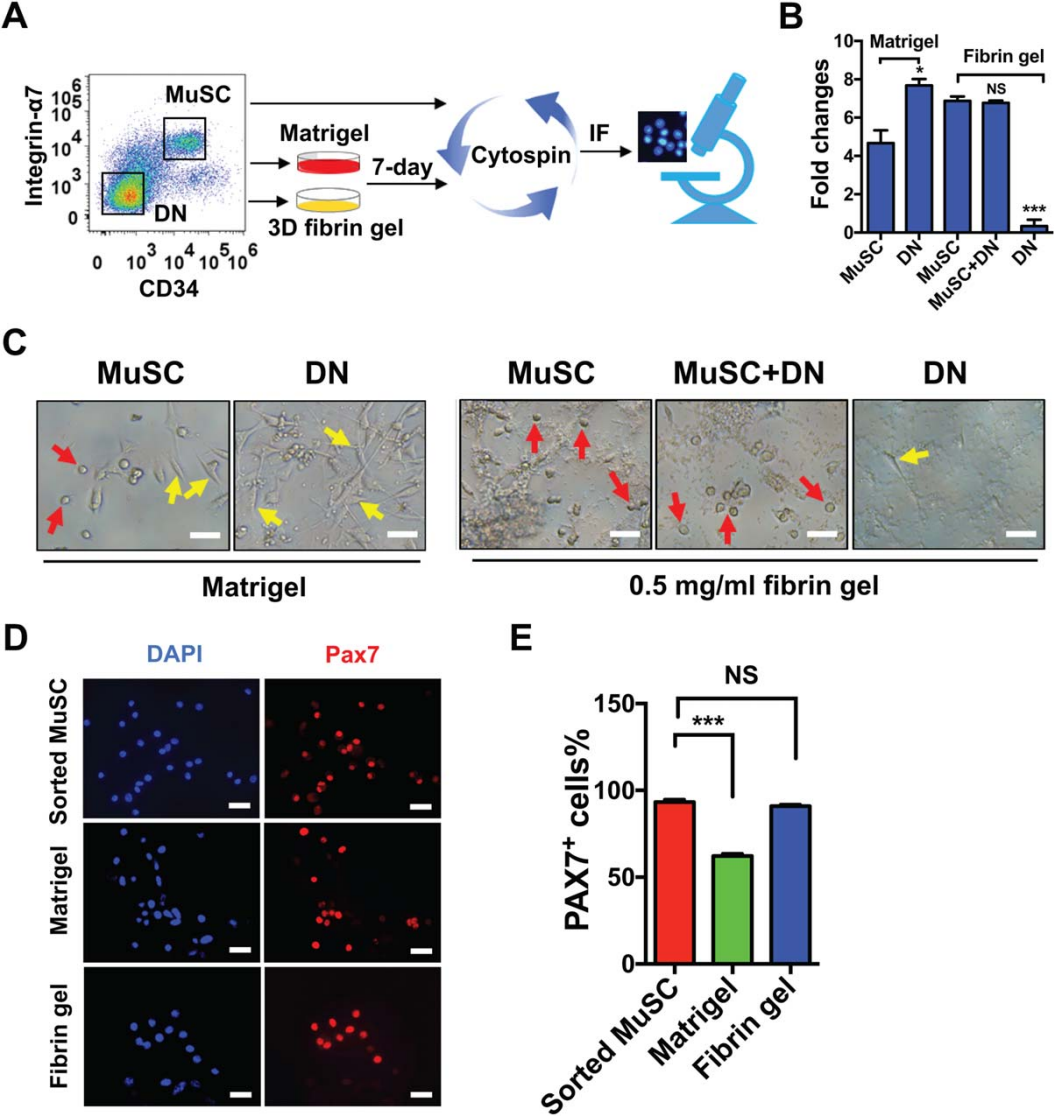
Differential growth potential of MuSCs and non‐MuSCs in three‐dimensional (3D) salmon fibrin gel. **(A)**: Schematic of ex vivo expansion of different subpopulations of skeletal muscle cells on Matrigel or in 3D salmon fibrin gel. Integrin‐α7^+^/CD34^+^ (MuSCs) and Integrin‐α7^−^/CD34^−^ (DN) cells in CD45^−^/CD11b^−^/CD31^−^/Sca1^−^ population were sorted by fluorescence‐activated cell sorting, and then cultured separately or in combination on Matrigel or in soft 3D salmon fibrin gels for 7 days. **(B)**: Fold increase in cell number of different cell subpopulations on Matrigel or in 3D salmon fibrin gel. Live cells were counted on day 7 of culture. Fold change is normalized to an initial seeding of 2,000 cells for each cell population. Error bars represent means ± SEM of three independent experiments. *, *p* < .05; ***, *p* < .001 versus sorted MuSCs in corresponding gel substrate, student's *t* test. NS, nonsignificant. **(C)**: Representative morphology of expanded cells. Sorted MuSCs and DN cells were cultured on Matrigel or separately or together in soft 3D fibrin gel for 7 days. Bright‐field images were then taken from the expanded progeny. Arrows in yellow and red displayed spindle‐shaped or round cell progeny, respectively. Scale bar, 50 μm. **(D)**: Immunofluorescence staining of MuSC marker PAX7 in progeny of sorted MuSCs expanded on Matrigel or in soft 3D fibrin gel after 7 days of culture. DAPI was used as nuclear counterstaining. Scale bar, 50 μm. **(E)**: Quantification of the proportion of PAX7^+^ cells in progeny of sorted MuSCs expanded on Matrigel or in soft 3D fibrin gel. Error bars represent means ± SEM of 3 independent experiments; ***, *p* < .001 versus sorted MuSCs, student's *t* test. Abbreviations: MuSCs, muscle stem cells; DAPI, 4′,6‐diamidino‐2‐phenylindole; DN, double‐negative.

To investigate whether 3D fibrin gel facilitates ex vivo expansion of MuSCs, we first sorted CD34^+^integrin‐α7^+^ (MuSCs) and CD34^−^integrin‐α7^−^ (double‐negative, DN) populations according to the established MuSC sorting protocols 11, 13. Matrigel is a gelatinous protein mixture that mainly consists of laminin, collagen IV, entactin, and heparin sulfate proteoglycan. It has been the most prevailing substrates used for culturing myogenic cells 39. Indeed, many previously established strategies improving ex‐vivo expansion of MuSCs were based on 2D culture on Matrigel 22, 24, 40. Therefore, it was selected as a standard to compare with the 3D soft fibrin gel in our study. Next, we plated the sorted MuSCs and DN cell populations separately or together on Matrigel‐coated plates or in soft 3D fibrin gel (Fig. 1A). After culture for 7 days, we found that the population of sorted MuSCs increased by fourfold on Matrigel and sevenfold in 3D fibrin gel (Fig. 1B). Interestingly, freshly sorted DN cells, which mainly consist of fibroblasts, thrived on Matrigel, but were difficult to maintain in soft 3D fibrin gel (Fig. 1B, 1C, Supporting Information Fig. S2). In agreement, a mixture of MuSCs and DN cells showed similar growth as MuSCs alone (Fig. 1B, 1C). In this experiment, we noticed both small round and spindle‐shaped cells in Matrigel‐expanded progeny. By contrast, 3D fibrin gel‐expanded progeny from either MuSCs or the mixture of MuSCs and DN population were dominated by small round cells (Fig. 1C).

To confirm the identity of progeny expanded from MuSCs on Matrigel or in 3D fibrin gels, we probed the cells for the expression of PAX7, a bona fide MuSC marker 41, and the differentiation marker Myogenin 7. The immunofluorescence (IF) staining showed that about 60% of MuSC‐derived cells expanded on Matrigel‐coated plates were positive for PAX7 expression, whereas over 90% of MuSC‐derived progeny in soft 3D fibrin gel retained PAX7 expression (Fig. 1D, 1E). In accordance, a significantly greater number of MuSC‐derived cells expressed the differentiation marker Myogenin when expanded on Matrigel versus soft 3D salmon fibrin gel (Supporting Information Fig. S3). Interestingly, the differentiation of satellite cells cultured in soft 3D salmon fibrin was delayed or inhibited, even after addition of differentiation medium (Supporting Information Fig. S4). Collectively, these data demonstrated that soft 3D salmon fibrin gel provides a unique milieu that favors the survival and proliferation of MuSCs while inhibiting their differentiation.

### Soft 3D Fibrin Gel Selectively Expands MuSCs from Bulk Skeletal Muscle Cells

**Figure 2:**
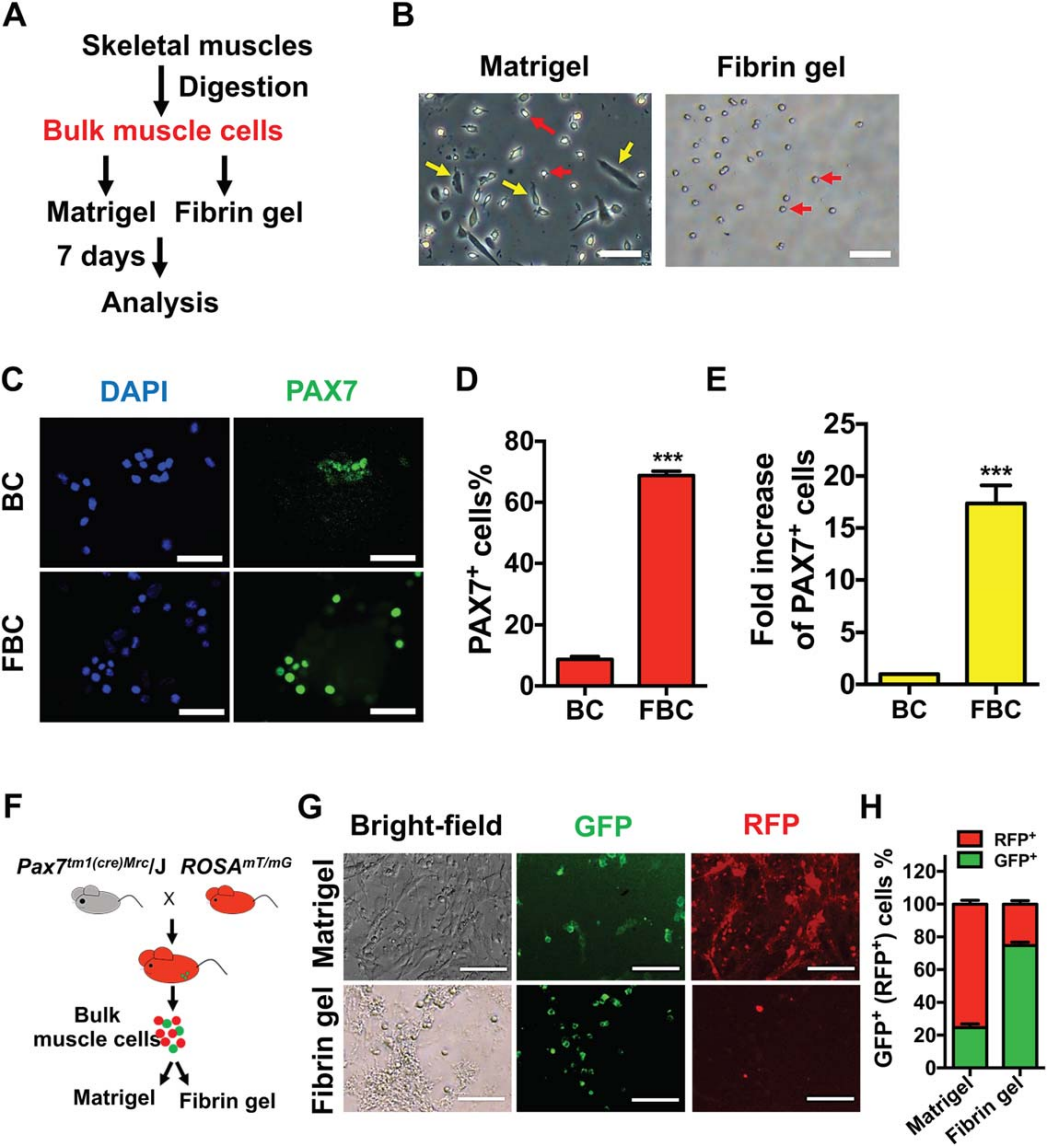
Soft three‐dimensional (3D) fibrin gel selectively expands skeletal muscle stem cells (MuSCs) from bulk muscle cells. **(A)**: Diagram for isolating and expanding bulk skeletal muscle cells. Skeletal muscle cells were isolated from C57BL/6 mice, and an initial of 5,000 bulk muscle cells were directly cultured on Matrigel or in soft 3D fibrin gel for 7 days. **(B)**: Representative photographs of bulk cell progeny expanded on Matrigel or in 3D soft fibrin gel (*n* = 4 independent experiments). Bright‐field images were taken on day 7 of culture. Arrows in yellow and red indicated spindle‐shaped and round cells, respectively. Scale bar, 100 μm. **(C)**: Coimmunostaining for PAX7 in fresh bulk skeletal muscle cells (BC) and their progeny expanded in fibrin gel (FBC) by day 7, with DAPI as nuclear counterstaining. Scale bar, 100 μm. **(D)**: Quantification of PAX7^+^ cell subpopulations in fresh bulk skeletal muscle cells (BC) and their progeny expanded for 7 days in soft 3D fibrin gel (FBC). Error bars represent means ± SEM of 3 independent experiments. ***, *p* < .001 versus fresh bulk cells, student's *t* test. **(E)**: Quantification of absolute PAX7^+^ cell number in initial 5000 primary bulk skeletal muscle cells (BC) and their progenies after 7‐day culture in fibrin gel (FBC). Error bars represent means ± SEM of 3 independent experiments. ***, *p* < .001 versus fresh bulk cells, student's *t* test. **(F)**: Experimental design for isolation and expansion of GFP‐labeled skeletal MuSCs. Bulk skeletal muscle cells were isolated from Pax7‐Switch mice (F1 offspring of *Pax7^tm1(cre)Mrc^* and *ROSA^mT/mG^* parental animals) whose MuSCs were specifically marked with membrane‐localized GFP while other non‐MuSCs were labeled with RFP. Freshly isolated bulk cells were cultured on Matrigel or in soft 3D fibrin gel for 7 days and then examined under fluorescent microscopy. **(G)**: Parallel phase and fluorescent micrographs of Matrigel‐ or soft fibrin gel‐expanded progeny of bulk muscle cells from Pax7‐Switch mice (*n* = 4∼5 mice). GFP^+^ cells indicated MuSCs and their derived cell lineages, while RFP‐expressing cells were non‐MuSCs. Scale bar, 100 μm. **(H)**: Quantification of the percentage of GFP^+^ and RFP^+^ progeny expanded on Matrigel or in soft 3D fibrin gel shown in (G). Error bars represent means ± SEM of 3 independent experiments. Abbreviations: BC, bulk cells; DAPI, 4′,6‐diamidino‐2‐phenylindole; FBC, fibrin gel‐expanded bulk cells; GFP, green fluorescent protein; RFP, red fluorescent protein.

Because of the scarcity of stem cells in adult muscle tissue and limited patient tissue, acquiring a sufficient starting number of pure MuSCs by FACS for ex vivo expansion is impractical for clinical therapy. Selective expansion of highly enriched adult stem cells from a small starting number of bulk cells would greatly advance stem cell‐based therapy in practice. Because soft 3D fibrin gels favorably supported the growth of MuSCs versus the non‐MuSC population (Fig. 1), we speculated whether MuSCs would be selectively expanded from bulk muscle cells when seeded in soft 3D fibrin gel. To address this question, we prepared bulk skeletal muscle cells and cultured an equal number of cells on Matrigel or in soft 3D fibrin gel (Fig. 2A). After 7 days of culture, spindle‐shaped cells exhibited an apparently superior growth over small round cells on Matrigel, while the vast majority of the expanded cells in soft 3D fibrin gel appeared round (Fig. 2B). IF staining showed that less than 10% of cells in primary bulk skeletal muscle cells express PAX7 (Fig. 2C, 2D). However, after 7 days of culture in soft 3D fibrin gel, greater than 70% of expanded cells were positive for PAX7 expression (Fig. 2C, 2D), which accounted for an over 15‐fold increase in number of PAX7^+^ cells (Fig. 2E).

To further confirm the selective expansion of MuSCs from bulk muscle cells in soft 3D fibrin gel using an in vivo genetic approach, we first generated “Pax7‐Switch” mice by crossing mice with Cre recombinase under the control of Pax7 promoter and regulatory elements (Pax7‐Cre knockin mice) 42 to mice harboring a dual‐color fluorescent reporter in the Rosa26 locus (mT/mG mice) 43. All the cells in Pax7‐Switch mice would express the membrane‐localized red‐fluorescent protein Tomato, with the exception of cells expressing Cre, which results in excision of Tomato and induction of membrane‐localized GFP expression. Thus, in Pax7‐Switch mice, MuSCs (Pax7^+^) express GFP while non‐MuSC populations express Tomato (Fig. 2F).

Next, we isolated bulk skeletal muscle cells from Pax7‐Switch mice and seeded them on Matrigel or in soft 3D fibrin gel. After 7 days of culture, we examined the morphology and expression of GFP and Tomato in the expanded cells. We found that morphologically round cells became the dominant cell type in fibrin gel and they concurrently expressed GFP (Fig. 2G, 2H). By contrast, there were only sporadic GFP^+^ round cells in Matrigel‐expanded progeny, with majority being Tomato^+^ spindle‐shaped (Fig. 2G, 2H). Collectively, these data indicated that soft 3D fibrin gel selectively expands MuSCs from bulk muscle cells without a need for sorting pure MuSC before culturing.

### Soft 3D Fibrin Gel‐Expanded MuSCs Are Transcriptionally Similar to Activated Satellite Cells

**Figure 3:**
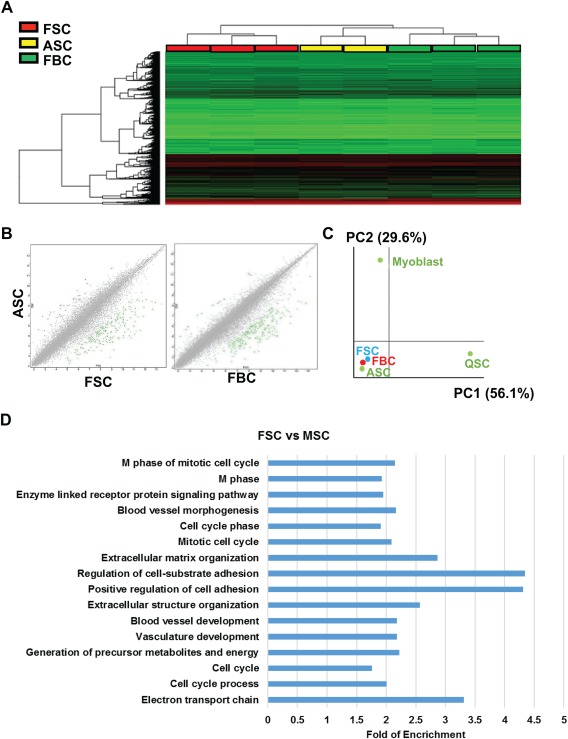
Muscle stem cells (MuSCs) expanded in soft three‐dimensional (3D) fibrin gel displayed similar gene expression profiles to ASCs. **(A)**: Heat map analysis of genome‐wide microarray gene expression profiles in fibrin gel‐expanded MuSCs. Sorted satellite cells (Integrin‐α7^+^/CD34^+^) and bulk skeletal muscle cells were cultured separately in soft 3D fibrin gel for 7 days. Total RNAs were then extracted from FSC and FBC, respectively, and used for microarray analysis. As a control, total RNA samples were extracted from ASC by a 48‐hour culture of quiescent satellite cells in growth medium in Matrigel‐coated wells. Triplicate RNA samples of each cell type were used for microarray analysis. **(B)**: Scatter plots showing transcriptome comparison of ASC versus FSC or FBC. **(C)**: Principal component analysis on identities of fibrin‐expanded cells. QSC, primary quiescent satellite cells; ASC, activated satellite cells by 48‐hours culture in growth medium; FSC, satellite cells expanded in fibrin gel for 7 days; Myoblast, satellite cells expanded on Matrigel for 7 days. **(D)**: Graphical representation of gene ontology (GO) enrichment within differentially expressed gene lists. Biological process derived from GO enrichment of differentially‐expressed genes in FSC at day 7 in comparison to MSC at day 7. Bars represent –log (*p* value) in each functional category. Functional GO enrichments were selected for significance by using a false discovery rate cutoff of 5%. Abbreviations: ASC, activated satellite cells; FBC, fibrin‐expanded bulk cells; FSC, fibrin‐expanded satellite cells; MSC, Matrigel‐cultured satellite cells.

To compare fibrin gel‐expanded MuSCs with primary ASCs, we used DNA microarrays to analyze the genome‐wide gene expression profiles of cell progeny expanded from the following three types of cells: (a) sorted satellite cells propagated in soft 3D fibrin gel for 7 days (FSC), (b) bulk skeletal cells cultured in soft fibrin gel for 7 days (FBC), (c) primary ASCs by culturing fresh satellite cells in growth medium for 48 hours (ASC). In comparison to ASCs, there were 685 (ASC vs. FSC, fold change >2 or < −2, ANOVA *p* value < .05) and 776 (ASC vs. FBC, fold change >2 or < −2, ANOVA *p* value < .05) genes among a total number of 34,472 genes differentially expressed in progeny from fibrin‐cultured satellite cells and bulk cells. The similarities of gene expression profiles were as high as 98.0% and 97.7%, respectively, (Fig. 3A, 3B). More importantly, none of the MuSC‐featured genes 7 were significantly different among the examined groups (Supporting Information Table S1), suggesting that fibrin gel‐expanded cells are highly enriched with activated MuSCs. Next, we applied PCA to analyze gene expression data and microarray data generated from quiescent satellite cells freshly sorted from resting skeletal muscle and myoblasts that were yielded by culturing satellite cells on Matrigel for 7 days. The quiescence or activation of satellite cells was confirmed by immunostaining on PAX7 and MyoD (Supporting Information Fig. S6). Our analyses demonstrated that fibrin‐expanded satellite cells and bulk cells fall closer to ASCs along the first (PC1) and second component (PC2) axis (Fig. 3C), suggesting that expanded MuSCs in soft 3D fibrin gel were transcriptionally similar to that of ASCs.

To gain further insight into what distinguishes satellite cell progeny expanded on Matrigel (MSC) and in soft 3D fibrin gel (FSC), we analyzed the molecular pathways enriched in genes induced in the FSC transcriptome relative to the MSC expression profiles. We found that several annotation groups such as cell adhesion, cell‐substrate adhesion as well as extracellular structure and matrix organization signaling pathways were significantly enriched in genes differentially regulated between the two groups (Fig. 3D). Taken together, these data showed that fibrin‐expanded progeny is identical to ASCs but largely distinct from Matrigel‐expanded myoblasts in transcriptome, which might be attributed to the completely different substrate mechanical property induced signal transduction.

### Fibrin‐Cultured MuSCs Robustly Differentiate In Vitro and Engraft Into Skeletal Muscle In Vivo

**Figure 4:**
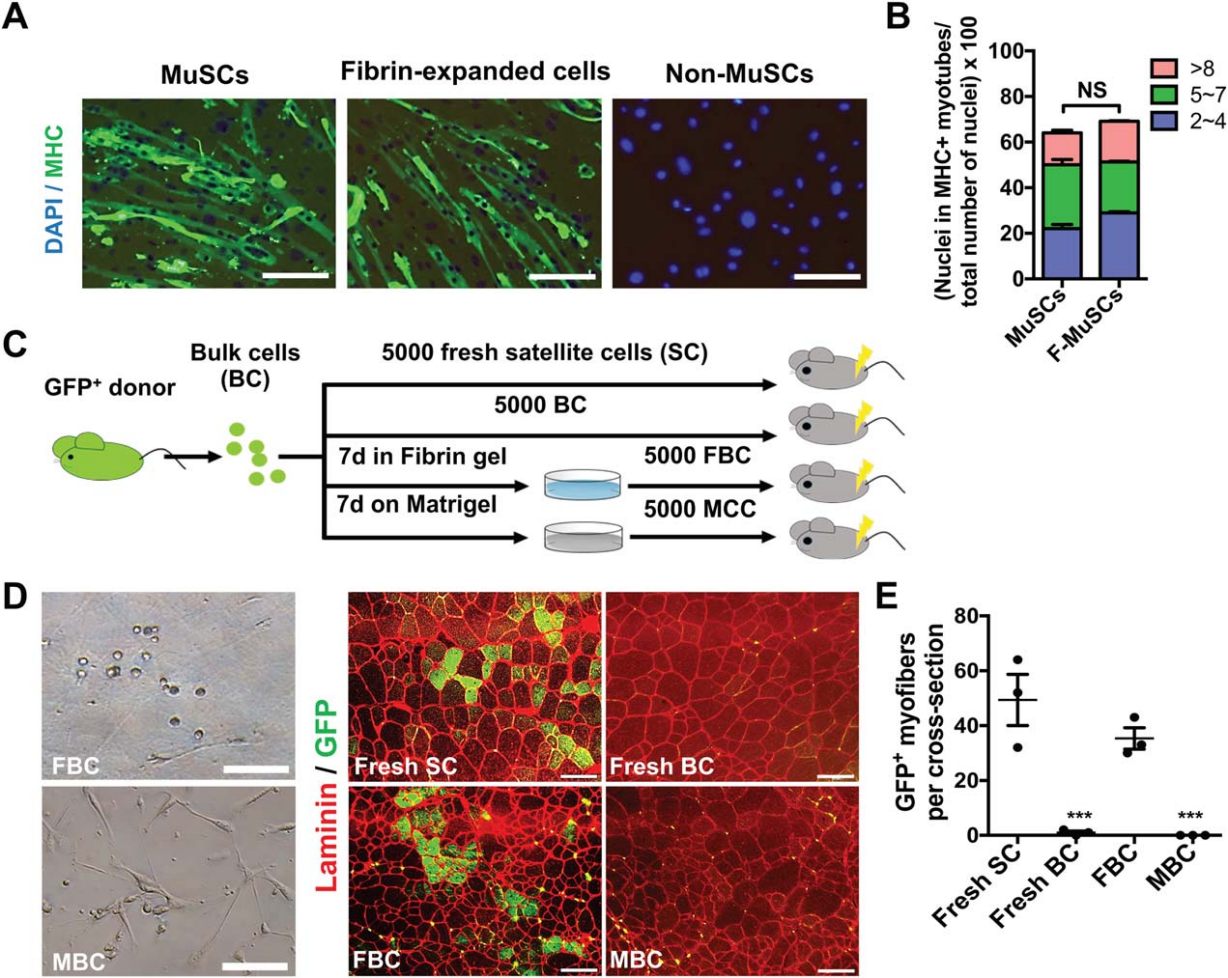
In vitro differentiation and in vivo engraftment potentials of fibrin gel‐expanded MuSCs. **(A)**: Immunostaining for differentiation marker MHC in in vitro differentiated muscle cells. An equal number of freshly sorted satellite cells and MuSCs expanded for 7 days from bulk muscle cells in soft 3D fibrin gel were plated on Matrigel‐coated plate and induced into differentiation in low serum condition for 3 days. Cell were then fixed and stained with anti‐MyHC antibody. Freshly sorted non‐MuSCs (Integrin‐α7^−^/CD34^−^) were induced as a negative control. Nuclei were stained with DAPI. Scale bar, 50 μm. **(B)**: Quantification of myonuclei in total nuclei from cells differentiated from freshly sorted satellite cells and fibrin‐expanded MuSCs. More than three random fields were chosen to count total nuclei and nuclei in MHC‐expressing myofibers. Error bars represent means ± SEM of 3 independent experiments. NS, nonsignificant. **(C)**: Experimental scheme for proving selective expansion of MuSCs from bulk muscle cells by soft 3D fibrin gel. At the start, 2,000 bulk skeletal muscle cells isolated from GFP‐transgenic donor were cultured in soft 3D fibrin gel or on Matrigel for 7 days. Five thousand of the expanded cells from either substrate were then transplanted separately into preinjured tibialis anterior (TA) muscles of NOD/SCID recipients. As controls, an equal number of fresh satellite cells or bulk muscle cells were directly transplanted into preinjured TA of NOD/SCID recipients. Engraftments of transplanted cells were assessed by immunostaining for GFP and laminin on frozen muscle sections 4 weeks after transplantation. **(D)**: Representative fluorescent micrographs of GFP‐expressing myofibers in frozen TA muscle sections of NOD/SCID recipient mice from (C) and photographs of bulk cell progeny expanded in 3D soft fibrin gel or on Matrigel. Laminin was costained in the sections and used to indicate the boundaries of myofibers. Scale bar, 100 μm. **(E)**: Quantification of the numbers of GFP‐expressing myofibers in TA muscles of recipient mice (*n* = 3 mice). Error bars represent means ± SEM. ***, *p* < .001 versus fresh SC‐transplanted mice, student's *t* test. Abbreviations: BC, bulk cells; FBC, fibrin gel‐expanded bulk cells; GFP, green fluorescent protein; MHC, myosin heavy chain; MBC, Matrigel‐expanded bulk cells; MuSCs, muscle stem cells; SC, satellite cells.

To confirm that soft 3D fibrin gel‐expanded MuSCs maintain stemness, we performed in vitro differentiation and transplantation assays. To assess in vitro differentiation capacity of fibrin gel‐expanded MuSCs, we first expanded satellite cells in soft 3D fibrin gel for 7 days, and then harvested and replated in Matrigel‐coated wells with differentiation culture medium. An equal number of freshly sorted MuSCs and DN population were included as a positive and negative control, respectively. Three days after in vitro differentiation, we determined the in vitro differentiation potential of fresh MuSCs and fibrin gel‐expanded MuSCs based on the expression of myosin heavy chain. Our results revealed that the differentiation capabilities of fibrin gel‐expanded MuSCs and freshly sorted MuSCs were comparable (Fig. 4A, 4B). Moreover, similar to those differentiated from fresh MuSCs, myotubes derived from fibrin gel‐expanded MuSCs were functionally contractile as well (Supporting Information videos). Together, these data demonstrated that fibrin gel‐expanded MuSCs possess robust differentiation potential in vitro.

Next, we assessed the engraftment capacity of fibrin gel‐selected MuSCs in vivo (Fig. 4C). We seeded an equal number of bulk skeletal muscle cells either in soft 3D fibrin gel or on Matrigel for 7 days. Then 5,000 expended progeny was transplanted into preinjured TA muscles of NOD/SCID recipients. For better assessing the selectivity and engraftment efficiency, we also transplanted an equal number of bulk muscle cells or fresh satellite cells as controls (Fig. 4C). As we showed above (Fig. 2B), fibrin‐expanded cells were uniformly round, while Matrigel‐propagated progeny included many spindle‐shaped fibroblasts prior to transplantation (Fig. 4D, left panel). Four weeks after transplantation, engraftment was examined microscopically by direct epi‐fluorescence in frozen muscle sections. As expected, neither bulk muscle cells nor cell mixtures expanded on Matrigel engrafted recipient mice. By contrast, fibrin gel‐expanded cells successfully engrafted recipient mice by presenting GFP^+^ fibers, and the efficiency was comparative to that of fresh satellite cells (Fig. 4D, 4E). Together, these results demonstrated that soft 3D fibrin gel indeed selectively expands functional MuSCs from bulk skeletal muscle cells.

### Fibrin Elasticity and Interaction Between Cell and Fibrin Gel Regulate Skeletal Muscle Cell Fates in Culture

**Figure 5:**
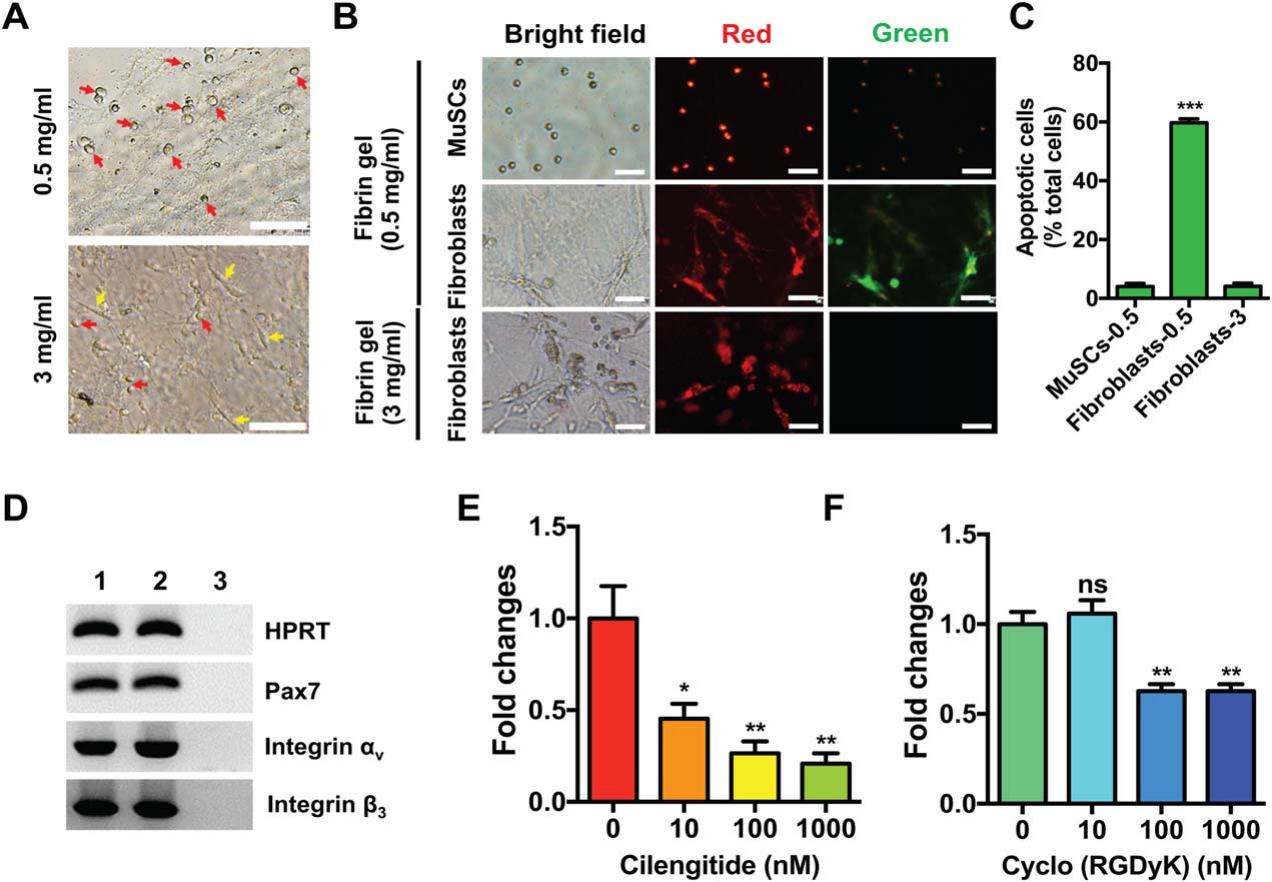
Elastic modulus and integrin‐engaged cell‐fibrin interaction regulate skeletal muscle cell fates. **(A)**: Representative bright‐field images of bulk skeletal muscle cells expanded for 7 days in 0.5 and 3 mg/ml fibrin gels. Arrows in yellow and red indicate spindle‐shaped and round cell progeny, respectively. Scale bar, 100 μm. **(B)**: Typical bright‐field and fluorescent micrographs of JC‐1 dye staining for MuSCs and muscle‐derived fibroblasts cultured in different concentration of fibrin gels. Sorted MuSCs and muscle‐derived fibroblasts were cultured in 0.5 and 3 mg/ml fibrin gels and stained with JC‐1 dye after 72 hours of culture. JC‐1 accumulates as aggregates (J‐aggregates) with intense red fluorescence in healthy cells with high transmembrane electrical potential (ΔΨ_M_), and remains as a monomer (J‐monomers) by exhibiting green fluorescence in the cytosol of apoptotic cells with low ΔΨ_M_. Scale bar, 50 μm. **(C)**: Quantification of apoptotic cells in MuSCs and muscle‐derived fibroblasts cultured in 0.5 and 3 mg/ml fibrin gels shown in (B). Error bars represent means ± SEM of 3 independent experiments. ***, *p* < .001 versus MuSCs cultured in 0.5 mg/ml fibrin gel group, student's *t* test. **(D)**: RT‐PCR analysis of α_v_β_3_ integrin expression in satellite cells (Integrin‐α7^+^/CD34^+^) (lane 1), and satellite cells expanded 7 days in soft three‐dimensional (3D) fibrin gel (lane 2). *Pax7* expression was examined to assess the purity of each cell type. A negative control (without cDNA template) was also included (lane 3). **(E, F)**: Quantification of fold changes in cell numbers of bulk skeletal muscle cells expanded for 7 days in soft 3D fibrin gel in the absence or presence of selective α_v_β_3_ integrin inhibitors Cilengitide (E) and Cyclo(RGDyK) (F). Error bars represent means ± SEM of 3 independent experiments. NS, nonsignificant; *, *p* < .05; **, *p* < .005 versus fibrin‐expanded cells in the absence of α_v_β_3_ integrin inhibitors group, student's *t* test. Abbreviation: MuSCs, muscle stem cells.

Our data showed that soft 3D fibrin gel selectively expands MuSCs in bulk muscle cells, and this selection was lost when the gel concentration increased (Fig. 5A, Supporting Information Fig. S1), suggesting an important role of substrate stiffness. Indeed, it has been reported that elastic stiffness, which is directly determined by the concentration of fibrin gel 44, affects cell morphology and behavior 45, 46. Nevertheless, that the stiffness of 0.5 mg/ml fibrin gel being favorable for selective expansion of MuSCs in the current study had not been determined before. In view of the advantage of atomic force microscopy (AFM) in measuring local stiffness over conventional rheometry which characterizes bulk material properties, we measured the stiffness of soft salmon fibrin in 0.5 mg/ml concentration by AFM. The elastic modulus of 0.5 mg/ml fibrin gel used in our studies was 470 ± 50 Pa from AFM measurement (Supporting Information Fig. S5).

To further delineate the molecular mechanisms of how soft 3D fibrin gel selectively expands MuSCs from bulk muscle cells, we assessed whether apoptosis played a role in the selective expansion of MuSCs versus non‐MuSCs in soft 3D fibrin gel. To do so, we seeded muscle‐derived fibroblasts in 0.5 and 3 mg/ml 3D fibrin gels, respectively. An equal number of sorted MuSCs were seeded in 0.5 mg/ml fibrin gel as a control. Seventy‐two hours after seeding the cells, we added JC‐1 dye 47, a mitochondrial membrane potential probe, into the medium and then examined apoptosis in cultured cells. As expected, progeny expanded from sorted MuSCs appeared round in 0.5 mg/ml fibrin gel and mainly displayed intense red fluorescent J‐aggregates, indicating their healthy status (Fig. 5B, 5C). However, fibroblasts grown in 0.5 mg/ml fibrin gel showed a high ratio of green to red fluorescence, implying a large proportion (∼60%) of cells were undergoing early apoptosis. By contrast, fibroblasts cultured in 3 mg/ml fibrin gel showed red fluorescence, indicating they were in a healthy status (Fig. 5B, 5C). Together, these results suggested that selective expansion of MuSCs by soft 3D fibrin gel was at least partially, if not totally, due to induction of apoptosis in fibroblasts.

Next, we investigated the molecular mechanisms by which MuSCs survive and expand in soft 3D fibrin gel. Previous studies have reported that the interactions between cell‐to‐cell and cell‐to‐extracellular matrix are mediated by a transmembrane receptor family of Integrins 48. Interestingly, the α_v_β_3_ integrin was reported to engage in the interaction between cells and fibrin gel 49, 50. Therefore, we examined the expression of α_v_β_3_ integrin in freshly sorted and fibrin‐expanded MuSCs by PCR. Our data showed that both α_v_ and β_3_ were expressed in fresh and fibrin‐expanded MuSCs (Fig. 5D), suggesting that α_v_β_3_ integrin might play a critical role in mediating the survival and expansion of MuSCs in fibrin gel. To assess the importance of α_v_β_3_ integrin, we blocked the interaction between α_v_β_3_ integrin on MuSCs with fibrin during culturing bulk muscle cells in soft 3D fibrin gel by adding gradient selective inhibitors Cyclo(RGDyK) or cilengitide into growth medium. After culturing for 7 days, we measured total cell numbers of expanded MuSCs. The results showed that blockage of α_v_β_3_ integrin binding with fibrin by either of the inhibitors significantly decreased the expansion of MuSCs in culture (Fig. 5E, 5F), indicating that the interaction between MuSCs and fibrin gel is critical for survival and proliferation of MuSCs.

Altogether, our results suggested a dual action model of how soft 3D fibrin gel selectively expands MuSCs from bulk muscle cells. In this model, the pliant elastic modulus of soft fibrin gel selectively caused apoptosis in muscle fibroblasts while α_v_β_3_ integrin enabled the survival and expansion of MuSCs in soft 3D fibrin gel, which in combination, contribute to the unique property of soft fibrin gel on selective expansion of MuSCs from bulk skeletal muscle cells.

## Discussion

Duchenne muscular dystrophy (DMD) is a lethal muscular degenerative disease caused by mutations in Dystrophin gene of X chromosome 51. Causes for muscle wasting in DMD was reported to be more than commonly acknowledged myofiber fragility, but also include intrinsically impaired MuSCs functions 52. Thus, strategies targeting MuSCs will provide more pertinent, promising and essential therapeutic approaches for DMD disease. Because of the extensive loss of regenerative capacity of cultured MuSCs, fresh satellite cells have been the most promising cell source for transplantation. In the past few years, several protocols have been established for isolation of fresh satellite cells from the skeletal muscles of adult mice by FACS 13, 15, 16, 53. To increase the purity, primarily sorted satellite cells were usually subjected to a secondary sorting 11, 13, which was inevitably at a cost of losing quantity of yield. More importantly, due to the scarcity of stem cells in adult muscle tissue and limited patient tissue, acquiring a sufficient starting number of fresh satellite cells by FACS for direct transplantation is impractical for clinical therapy. Soft 3D salmon fibrin gel culture system demonstrated in this study could selectively propagate MuSCs with highly reserved regenerative competency from a small starting number of bulk skeletal muscle cells. Our data showed that fibrin‐expanded MuSCs and fresh satellite cells have comparable engraftment efficiency. Thus, this convenient culture system solves the bottleneck of application of adult MuSCs by providing sufficient number of competent myogenic cells, thus strongly advancing MuSCs‐based gene therapies for treatment of genetic muscle disorders.

We showed in our study that soft 3D fibrin gel supported the growth of fresh satellite cells, partially through fibrin‐α_v_β_3_ integrin interaction. At least seven specific cell‐binding domains in the fibrin molecule have been reported to mediate interactions between fibrin and hematopoietic cells 54. However, binding sites in fibrin that interact with myogenic cells remain largely unknown. Previous study demonstrated that human newborn aortic smooth muscle cells could adhere to fibrin clots through integrin α_v_β_3_ 55. We have confirmed the expression of α_v_β_3_ integrin in satellite cells before and after fibrin gel culture. Cyclo(RGDyK) and Cilengitide used in our study are potent and selective α_v_β_3_ integrin inhibitors. Therefore, we propose that impaired and decreased adhesion of MuSCs to fibrin by α_v_β_3_ integrin inhibitors was responsible in certain degree for the compromised cell growth in soft 3D fibrin gel.

Result from our AFM measurement showed that the elastic modulus of 0.5 mg/ml fibrin gel is approximately 470 ± 50 Pa, which was seemingly unfit for the growth of MuSCs because it was about four orders of magnitude softer than in vivo mechanical properties of muscle niche that favors MuSC self‐renewal 21. However, we believe that the unique property of nonlinear elasticity of fibrin gel might have played an important role in accommodating MuSCs survival and expansion 36, 44. Nonlinear elasticity endows filamentous fibrin gel simultaneously the property of strain stiffening, which is rarely seen in synthetic polymers 56. Indeed, it has been shown that the modulus of 60 Pa fibrin gel at 80% strain was as high as 3.7 kPa in reality 44. Therefore, it is conceivable that MuSCs actively deform soft fibrin gel to sense high strain modulus rather than low strain modulus of the substrate. This characteristic may also explain an abrupt loss of selectivity on round MuSCs when fibrinogen concentration increases from 0.5 to 1 mg/ml in our studies.

There are many methods for preservation of the regenerative capability of ex vivo cultured MuSCs, but all require seeding sorted satellite cells in starting cultures. Compared to other hydrogels, fibrin gel demonstrated the most attractive property of cell growth selection. Soft fibrin gel was previously reported to promote selection and growth of cancer stem cells from pooled heterogeneous cancer cells as well 57, but the mechanism was not clear. Freshly prepared bulk skeletal muscle cells are such a mixture containing skeletal muscle cells, connective tissue fibroblasts, vascular endothelial cells, hematopoietic cells, macrophages, and so forth. Growth medium used for expansion of satellite cells nourishes fibroblasts but lacks many essentials for many other types of cells. For example, in addition to basic medium, culture of vascular endothelial cells requires vascular endothelial growth factor, pituitary glands‐derived endothelial cells growth supplement, heparin, EGF, and hydrocortisone 58. Besides the demanding requirements on culture medium, cells like hematopoietic cells are anchorage‐independent. They could be easily removed during the routine medium changes. Instead, under myogenic cell culture condition fibroblasts could grow up to 99% of the total population after 2 weeks 59. Therefore, fibroblast is the major source of contamination for skeletal muscle cell culture. The effect of mechanical tension on the induction of apoptosis in fibroblasts during wound healing process has been described elsewhere by Grinnell et al. 60. It turned out that a softer elasticity favors MuSCs expansion but induces apoptosis in fibroblasts. Such a property of soft 3D fibrin gel is especially valuable for advancing the intervention of effective stem cell‐based therapies.

Our results indicated that soft 3D fibrin gel holds a competency of delaying loss of the MuSC properties of expanded MuSCs in comparison to Matrigel (Supporting Information Figs. S3, S4). Gene set enrichment analysis of the most differentially regulated genes between 7‐day‐fibrin‐ and Matrigel‐expanded satellite cells demonstrated marked alteration in expression levels of genes related to cell adhesion, cell‐substrate adhesion as well as extracellular structure and matrix organization signaling pathways (Fig. 3D), indicating diverse adaptations to different substrates of fibrin gel and Matrigel. Particularly, *Itga6* 61, 62 and *B4galt1* [62], two genes specific for myogenic differentiation 62, were also upregulated in Matrigel‐expanded satellite cells. Substrate rigidity modifies the activation of integrin clustering, which is considered as the initial step in subsequent signal transduction in stem cell genomic regulation 63. One of the most important integrin‐mediated adhesions involved in mechanotransduction is focal adhesion (FA) 64. Previous studies reported that the mechanical force exerted on cells promotes FA formation and induce force‐mediated stem cell differentiation 65. In support of this mechanism, we also detected numerous significantly upregulated FA constituent and regulatory genes in Matrigel‐expanded satellite cells. Therefore, we believe that the different cell fates in fibrin‐ and Matrigel‐expanded MuSCs were highly affected by their completely distinct mechanical properties.

Nevertheless, physical cues alone can hardly recapitulate the maximal function of cultured adult stem cells 21. Stem cell niche is a dynamic and complex environment filled with biophysical, mechanical and biochemical properties specific for each tissue. Full retention of stem cell properties demands both physical and biochemical cues. To date, a number of signaling molecules have been reported to regulate MuSC quiescence 66, self‐renewal 23, 67, division 22, proliferation 26, 68, 69, and differentiation 70, 71, 72. Our unpublished data demonstrated that many of these molecules significantly enhanced propagation of MuSCs in soft 3D fibrin gel without affecting its selective property. Therefore, it is tempting to identify combinations of myogenic growth factors and small molecules that can significantly enhance selective expansion of MuSCs from bulk muscle cells in soft 3D fibrin gel to further advance the intervention of effective stem cell‐based muscular disease therapy.

## Conclusion

In this study, we developed a robust 3D fibrin gel culture platform that could selectively expand primary skeletal MuSCs from bulk muscle cells without the need for cell sorting. Fibrin gel‐expanded MuSCs are transcriptionally similar to ASCs and maintain regenerative capacity both in vitro and in vivo. This culture system should enable customized genome editing via CRISPR/Cas9 technology to correct mutations in disease‐causing genes in MuSCs. Thus, our studies paved the way for developing safer, more convenient, and robust MuSC‐based gene therapies for treating genetic muscular disease.

## Author Contributions

P.Z. and W.S.W.: conception and design, collection and/or assembly of data, data analysis and interpretation, manuscript writing and revision, final approval of manuscript; W.S.W.: financial support; F.W., X.W., and G.S.: collection and/or assembly of data, final approval of manuscript; Y.Z.: data analysis and interpretation, final approval of manuscript; J.M.: manuscript editing and final approval of manuscript. Y.Z. and F.W.: contribute equally to this article.

## Disclosure of Potential Conflicts of Interest

The authors indicated no potential conflicts of interest.

## Supporting information

Supporting Information

Supporting Information

Supporting Information
