# Supplementary material for: Selective Expansion of Skeletal Muscle Stem Cells from Bulk Muscle Cells in Soft Three‐Dimensional Fibrin Gel
Source: Stem Cells Transl Med. 2017 Feb 28;6(5):1412–23. doi: 10.1002/sctm.16-0427 (PMC5442710; doi:10.1002/sctm.16-0427)
Supplement: Supplementary file 1 — Supporting Information [file SCT3-6-1412-s001.pdf]

Supplementary

Selective Expansion of Myogenic Progenitor Cells from Bulk Skeletal Muscle Cells in Soft 3D Fibrin Gel

Pei Zhu, Yalu Zhou, Furen Wu, Yuanfang Hong, Xin Wang, Gajendra Shekhawat, Jeffrey Mosenson, and Wen-Shu Wu

Supplementary Materials and Methods

Supplementary Figures:

Figure S1: Growth of Early Passage Mouse Primary Myoblasts on Matrigel and in Soft 3D Salmon Fibrin Gel

Figure S2: Selective Growth of MuSCs in Soft 3D Salmon Fibrin Gel

Figure 3S: Differentiation Prohibition of MuSCs in Soft 3D Fibrin Gel

Figure S4: Induced Differentiation of MuSCs is Impaired in Soft 3D Fibrin Gel

Figure S5: Mechanical Characterization on Soft Fibrin Gel Using AFM

Figure S6: Immunofluorescence of quiescent satellite cells (QSC) and myoblasts for myogenic markers

Figure S7: Immunohistochemistry on GFP-expressing myofibers in frozen TA muscle sections of NOD/SCID recipient mice

Supplementary Tables:

Table S1: Microarray Analysis of MuSC-featured Genes in Culture Reactivated Satellite Cells (ASC), Fibrin-expanded Sorted Satellite Cells (FSC), and Fibrin-expanded bulk Skeletal Muscle Cells (FBC)

Table S2: Primer Sequences

Supplementary Videos:

Video S1: Contraction of Myofibers Derived from MuSCs Expanded by Soft 3D Fibrin Gel

Video S2: Contraction of Myofibers Derived from Freshly Sorted MuSCs

SUPPLEMANTARY MATERIALS AND METHODS

Histology and Immunohistochemistry in Muscle Cryosections

Fresh TA muscles were embedded in Tissue-Tek® O.C.T™ compound (Fisher), and frozen in liquid nitrogen cooled isopentane, and stored at -80°C until analysis. Frozen muscles were cross-sectioned (10 µm) using a Leica CM1850 cryostat. For immunohistochemistry study, air-dried muscle sections were fixed with 4% paraformaldehyde (PFA) and permeabilized in 0.2% Trition X-100. Tissue sections were then blocked in phosphate buffer saline (PBS) with 5% goat serum, 2% bovine serum albumin and 1% Tween- 20 for 1h and followed by incubation with primary antibodies overnight at 4°C. Rat anti-laminin (clone A5, Pierce, 1:100 dilution) and mouse anti-dystrophin (Sigma, 1:100 dilution) antibodies were used to denote myofiber boundaries and indicate restoration of dystrophin protein, respectively. GFP was detected microscopically by epifluorescence. After washing with PBS, sections were then incubated with Alexa-conjugated secondary antibodies (Invitrogen, 1:200 dilution).

Immunofluorescence Staining

Cell were cytospun onto the slides and fixed with 4% PFA. After permeabilization by 0.2% Trition X-100, cells were then blocked in PBS with 1% bovine serum albumin (BSA) for 1h at room temperature followed by staining with primary antibody overnight at 4°C. Cells were washed three times with PBS and incubated with secondary antibody for 1h at room temperature. Nuclei were labeled with DAPI. Primary antibodies used were mouse anti-Pax7 (1:10, DSHB), mouse anti-Myogenin (1:20, DSHB), mouse anti-myosin heavy chain (1:20, DSHB) and rabbit anti-ZsGreen (1:100, Clontech).

RNA Extraction and RT-PCR

Total RNA was extracted from muscle cells using TRIzol Reagent (life technologies). First strand cDNA synthesis was performed using RT^{TM} Master Mix (LAMDA BIOTECH). Quantitative real-time PCR (qPCR) was performed using GoTaq® Green Master Mix (Promega) on Bio-Rad C1000Touch Thermal Cycler. The primer sequences for PCR are listed in Table S2.

Apoptosis Assay

Early apoptosis was determined by assessing mitochondrial health using 5,5',6,6'tetrachloro-1,1',3,3'-tetraethylbenzimidazol-carbocyanine iodide (JC-1) Mitochondrial Membrane Potential Assay Kit (Cayman Chemical). Briefly, sorted satellite cells or muscle-derived fibroblasts were cultured in 0.5 and/or 3 mg/ml salmon fibrin gel. Seventy-two hours after seeding, JC-1 staining solution (100 µl per 1 ml of growth medium) was added into each well, and incubated in a CO_2 incubator at 37°C for at least 30 min. Cells were then analyzed directly in the culture medium using fluorescent microscopy. Healthy cells with mainly JC-1 J-aggregates was detected with fluorescence settings detecting Texas Red (excitation/emission = 590/610 nm). Apoptotic or unhealthy cells with mainly JC-1 monomers was detected with fluorescence settings detecting FITC (excitation/emission = 485/535 nm).

Measurement of Fibrin Gel Stiffness

Atomic force microscopy indentation was used to characterize the mechanical properties of fibril gel in growth medium. To reduce concentrated stress on fibril gel samples during AFM mechanical measurement, SiO_2 bead with a diameter of ~ 850 nm attached to a tipless silicon nitride triangular cantilever was adopted for elasticity measurements with 30 nm gold coating (Novascan Technologies). The deflection sensitivity was calibrated by repeated contact mode indentation on a clean glass slide (VWR International, Inc.) and the spring constant of the compliant AFM cantilever was measured to be k = 0.06 Nm^{-1} by thermal noise method in liquid [2]. The tip radius was determined post-mortem by scanning electron microscope (SEM, Hitachi SU8030). Elastic modulus was measured by fitting the experimental data to the spherical Hertzian contact mechanical model [3]. A home-made MATLAB (MathWorks, Inc.) code was used to analyze the experimental data, where the goodness values R^2 exceeded 0.85 in all curve fittings as reported in [4]. The measurements were repeated at 3 various positions with 10 measurements at each position.

SUPPLEMENTAL REFERENCES

1. Sacco A, Doyonnas R, Kraft P et al. Self-renewal and expansion of single transplanted muscle stem cells. **Nature**. 2008;456:502-506.
2. Hutter JL, Bechhoefer J. Calibration of Atomic-Force Microscope Tips (Vol 64, Pg 1868, 1993). **Rev Sci Instrum**. 1993;64:3342-3342.
3. Sneddon IN. The relation between load and penetration in the axisymmetric boussinesq problem for a punch of arbitrary profile. **International Journal of Engineering Science**. 1965;3:47-57.
4. Wang X, Bleher R, Brown ME et al. Nano-Biomechanical Study of Spatio-Temporal Cytoskeleton Rearrangements that Determine Subcellular Mechanical Properties and Endothelial Permeability. **Sci Rep-Uk**. 2015;5.

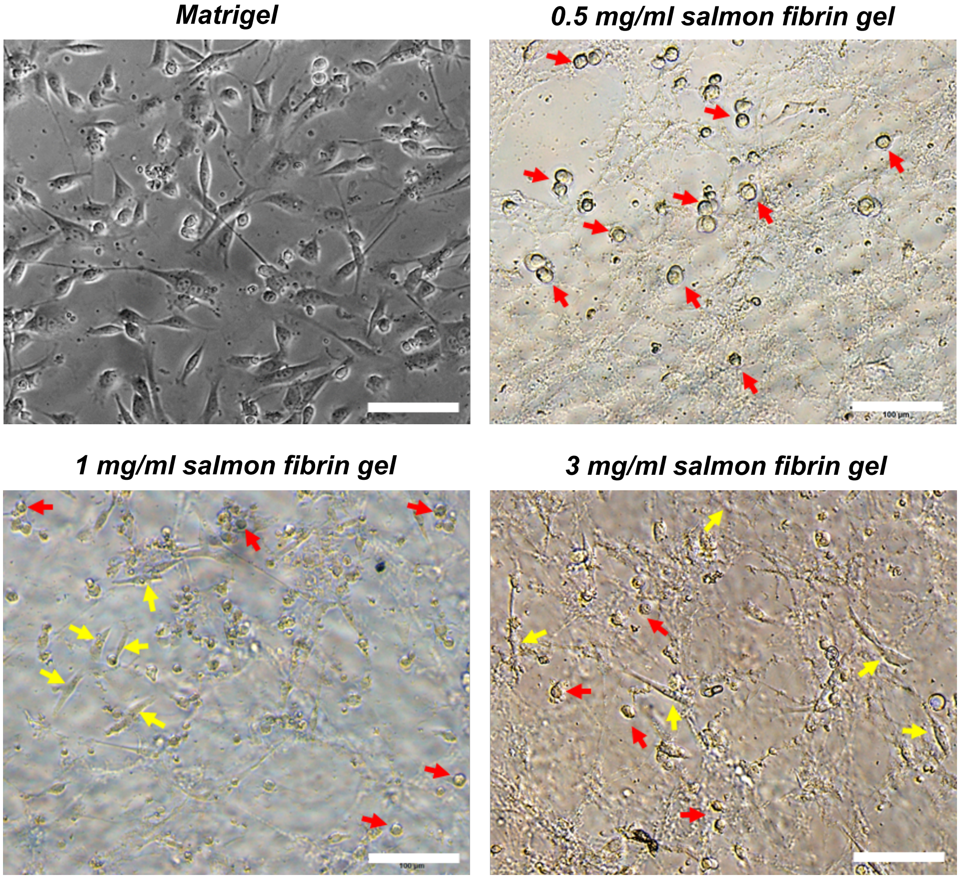

Figure S1: Growth of Early Passage Mouse Primary Myoblasts on Matrigel and in Soft 3D Salmon Fibrin Gel, Related to Figure 1, 4

Bulk skeletal muscle cells were isolated from hindlimb muscles of C57BL/6 mice, and then serially pre-plated in 10-cm culture dish to enrich myoblasts and remove most of fibroblasts. These early passage cells were separately seeded on Matrigel-coated plate or in different concentrations of 3D salmon fibrin gels. Phase contrast images were taken at day 7 of culture. Arrows in yellow and red displayed spindle-shaped and round cell progeny, respectively. Scale bar, 100 µm.

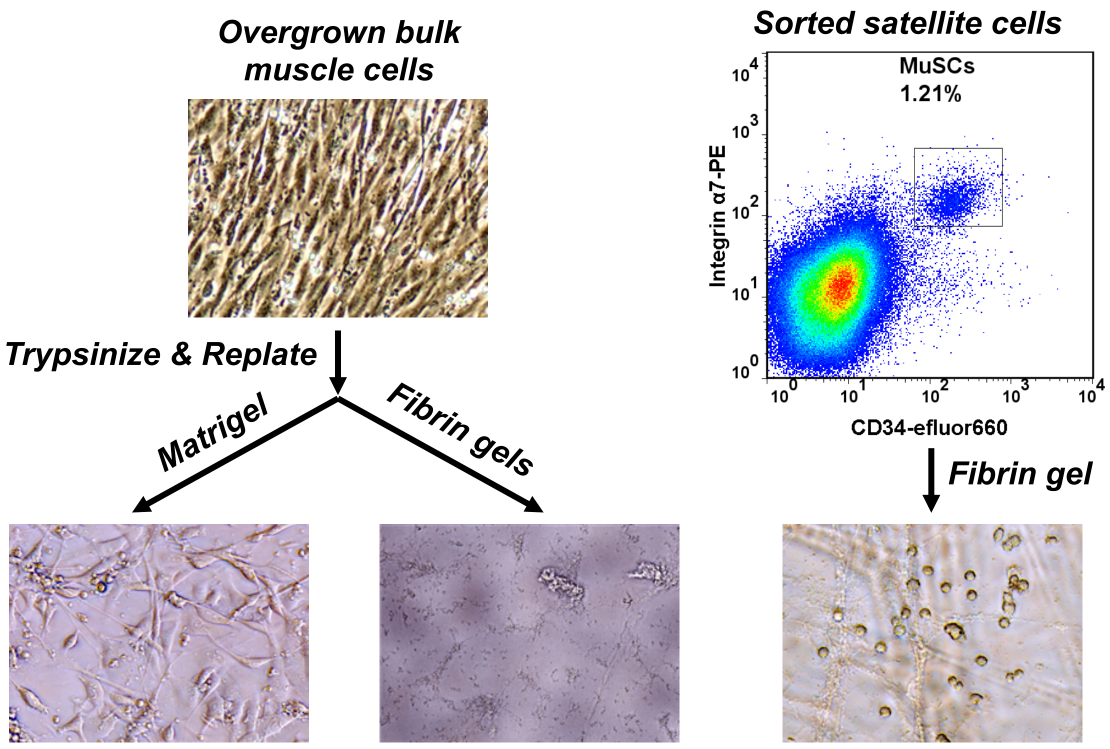

Figure S2: Selective Growth of MuSCs in Soft 3D Salmon Fibrin Gel, Related to Figure 1

Bulk skeletal muscle cells were isolated from hindlimb muscles of C57BL/6 mice, and then cultured in Matrigel-coated plate with fibroblast-favored DMEM/10% FBS growth medium (left panels). Cells were allowed to overgrown to reach confluence. The resultant fibroblasts and myocytes were then trypsinized and re-plated on Matrigel-coated plate or in 0.5 mg/ml 3D salmon fibrin gel. MuSCs ($CD45^-/CD11b^-/CD31^-/Sca1^-$/integrin-$\alpha7^+/CD34^+$) in bulk skeletal muscle cells were sorted by FACS, and then cultured in 0.5 mg/ml 3D salmon fibrin gel (right panels). Representative phase contrast images were taken by day 7 of culture in each growth condition (**n** = 3). Cells were visualized at x10 magnification using an Olympus CKX41 fluorescence microscope.

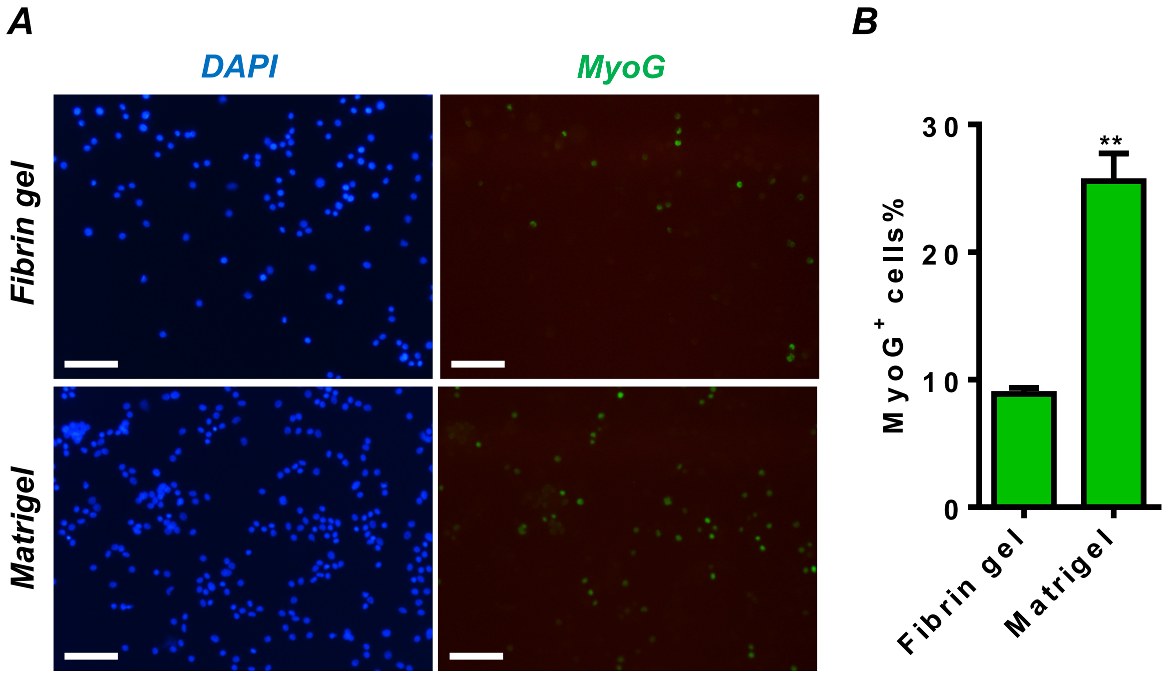

Figure S3: Differentiation Prohibition of MuSCs in Soft 3D Fibrin Gel, Related to Figure 1
(***A***) Immunofluorescence staining of differentiation marker Myogenin (MyoG) in progeny of sorted MuSCs expanded on Matrigel or in soft 3D fibrin gel for 7 days. DAPI was used as nuclear counterstaining. Scale bar, 100 μm.
(***B***) Quantification of the proportion of MyoG$^+$ cells in progeny of freshly sorted MuSCs expanded on Matrigel or in soft 3D fibrin gel. Error bars represent means ë SEM from n = 3 independent experiments. ** $p < 0.005$, student's **t**-test.

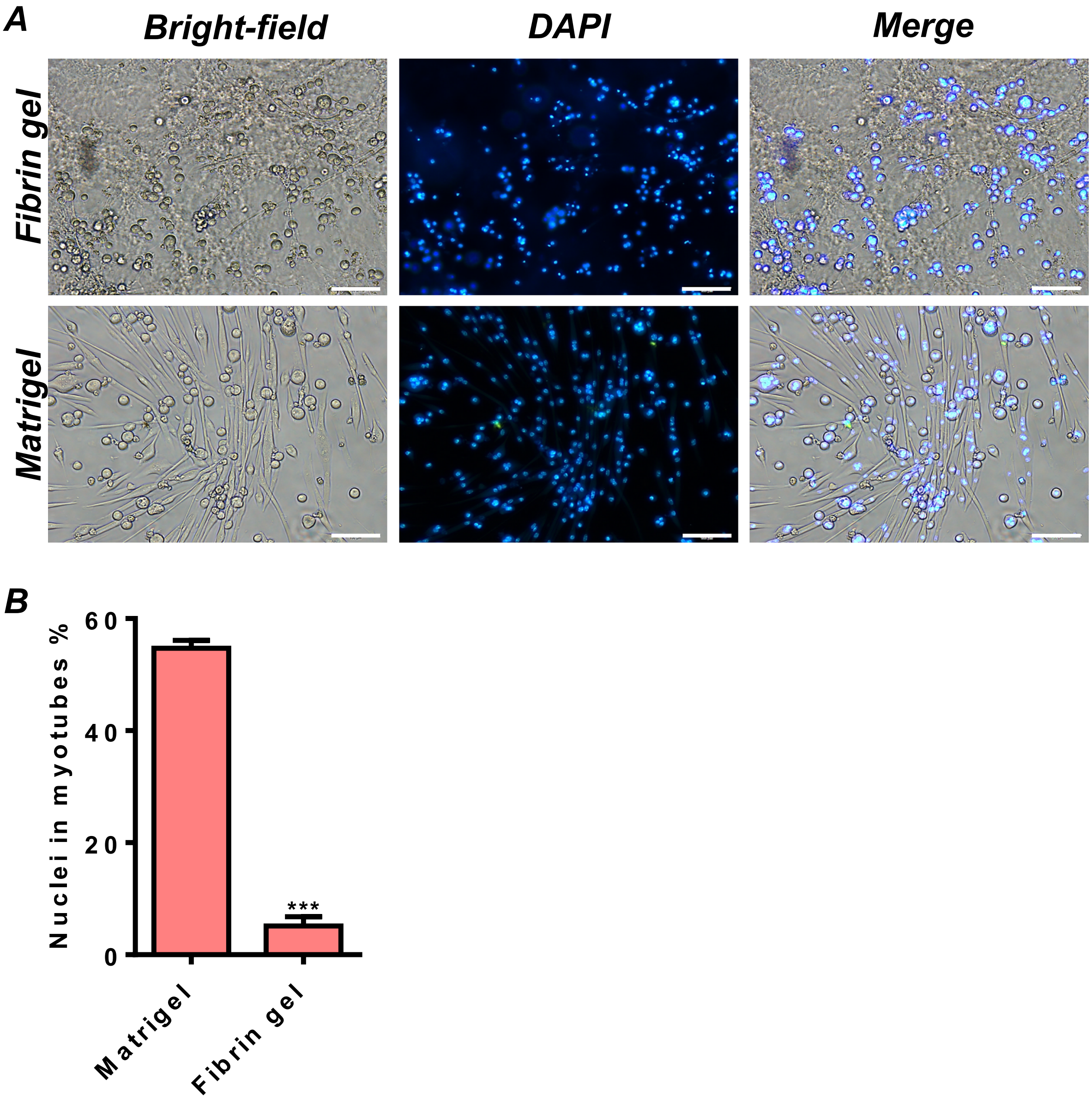

Figure S4: Induced Differentiation of MuSCs is Impaired in Soft 3D Fibrin Gel, Related to Figure 1

Fresh satellite cells were seeded separately in soft 3D fibrin gel or on Matrigel, and induced into differentiation in DMEM supplemented with 5% horse serum. By day 5 post induction, cells were fixed with 4% paraformaldehyde and stained with DAPI.

(***A***) Bright-field images taken at day 5 post induction of differentiation. Nuclei were stained with DAPI. Bar, 100 µm.

(B) Quantification of fusion index by calculating percentage of nuclei within myotubes using an Image J software. Error bars represent means ë SEM from n = 3 independent experiments. ***, $p < 0.001$, student's **t**-test.

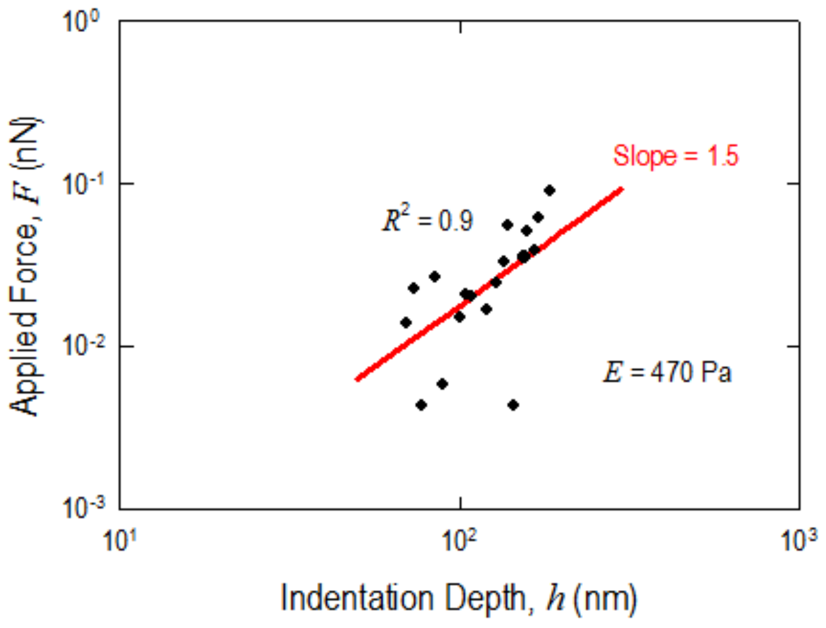

Hertz Contact Theory:

$$\mathbf{F} = 4/3.\ \mathbf{E}/(1\text{-}n^2).\mathbf{R}^{1/2}\mathbf{h}^{3/2}$$

F: Applied compressive load
R: Sphere radius
h: Indentation depth

Figure S5: Mechanical Characterization on Soft Fibrin Gel Using AFM, Related to Figure 4

Atomic force microscopy indentation was used to characterize the mechanical properties of 0.5 mg/ml fibril gel in growth medium. Elastic modulus was measured by fitting the experimental data to the spherical Hertzian contact mechanical model.

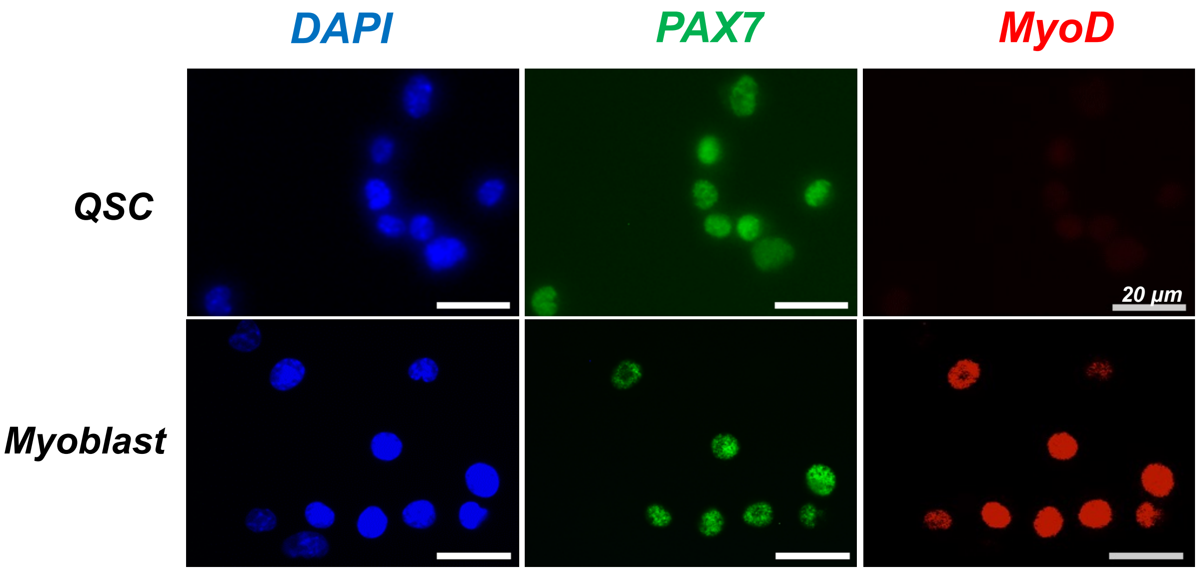

Figure S6: Immunofluorescence of quiescent satellite cells (QSC) and myoblasts for myogenic markers, Related to Figure 3

Quiescent muscle stem cells were isolated from resting skeletal muscles by careful gating of CD45-/CD11b-/CD31-/Sca1-/Integrin-צ7+/CD34+ population in total mononuclear cells, while myoblasts were prepared by growing satellite cells on Matrigel-coated plate for 7 days. Cells were stained with PAX7 (green) and MyoD (red) antibodies.

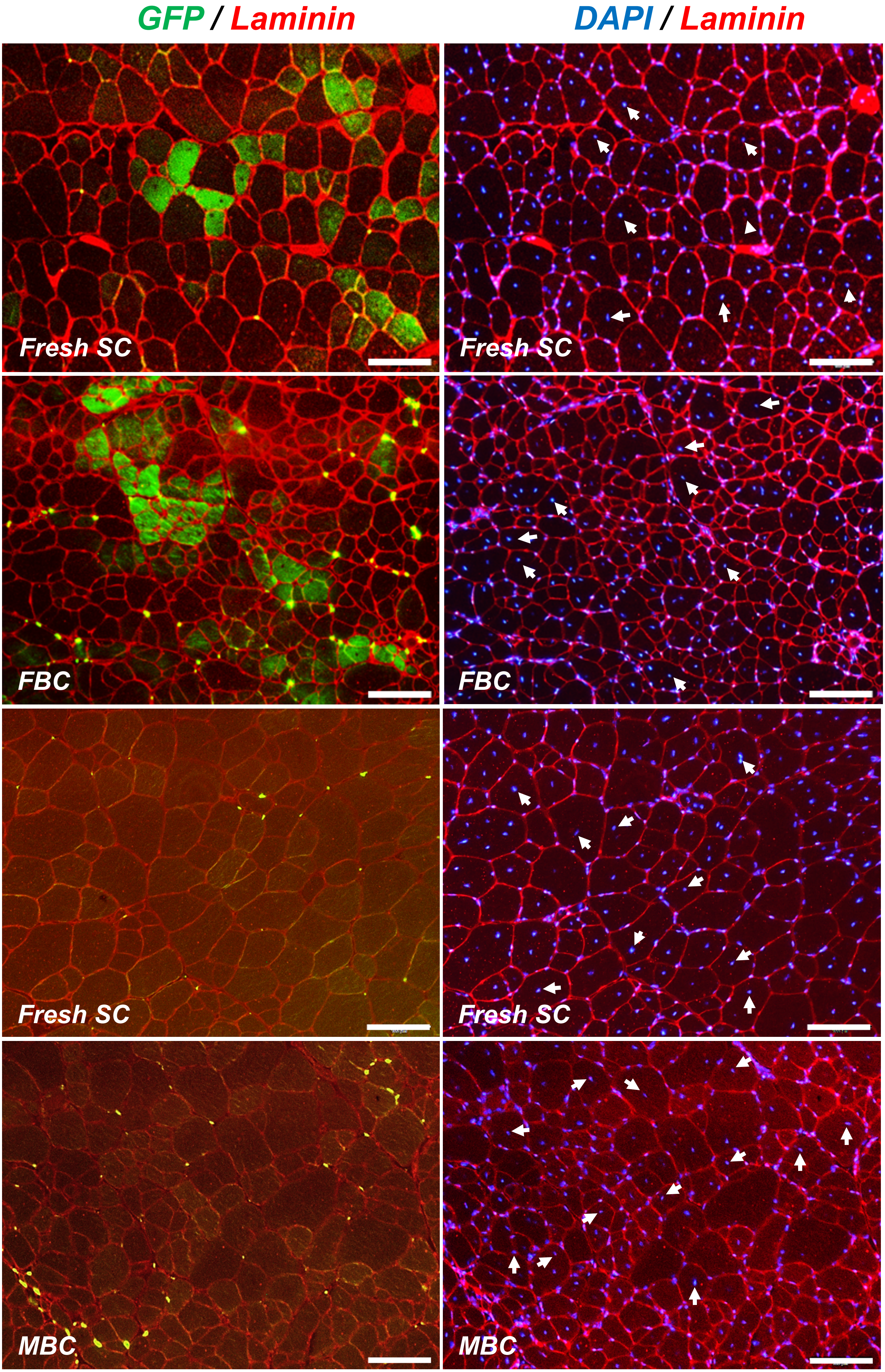
GFP / Laminin
DAPI / Laminin
Fresh SC
Fresh SC
FBC
FBC
Fresh SC
Fresh SC
MBC
MBC

Figure S7: Immunohistochemistry on GFP-expressing myofibers in frozen TA muscle sections of NOD/SCID recipient mice, Related to Figure 4

2000 bulk skeletal muscle cells isolated from GFP-transgenic donor were cultured in soft 3D fibrin gel or on Matrigel for 7 days. Five thousand of the expanded cells from either substrate were then transplanted separately into pre-injured TA muscles of NOD/SCID recipients. As controls, an equal number of fresh satellite cells or bulk muscle cells were directly transplanted into pre-injured TA of NOD/SCID recipients. Engraftments of transplanted cells were assessed by immunostaining on GFP and laminin on frozen muscle sections 4 weeks after transplantation. For each cell type-transplanted group, left and right panels showed exactly the same view on the muscle section, with the left panel displaying overlapping of GFP (green) and Laminin (red) channels while the right one displaying DAPI (blue) and Laminin (red) channels, respectively.

Transcript Cluster ID	ASC Bi-weight Avg Signal (log2)	FSC Bi-weight Avg Signal (log2)	FBC Bi-weight Avg Signal (log2)	A p-value (All Con	value (All Cor	Gene Symbol	Description
17464549	4.06	4.07	4.01	1	1	Calcr	calcitonin receptor
17456161	7.51	6.45	7.38	1	1	Cav1	caveolin 1, caveolae protein
17221014	7.15	5.73	10.44	1	1	Cd34	CD34 antigen
17506460	7.73	8.74	7.73	1	1	Cdh15	cadherin 15
17226593	9.41	10.24	9.27	1	1	Cxcr4	chemokine (C-X-C motif) receptor 4
17535752	9.41	8.92	9.03	1	1	Emd	emerin
17213265	6.14	6.38	6.51	1	1	Fzd7	frizzled homolog 7 (Drosophila)
17238605	10.54	10.72	9.88	1	1	Itga7	integrin alpha 7
17507014	9.95	9.84	10.32	1	1	Itgb1	integrin beta 1 (fibronectin receptor beta)
17406783	10.75	10.88	11.05	1	1	Lmna	lamin A
17456176	10.85	9.06	8.55	1	1	Met	met proto-oncogene
17244726	8.07	7.56	6.54	1	1	Myf5	myogenic factor 5
17478175	11.49	11.4	10.85	1	1	Myod1	myogenic differentiation 1
17526776	10.25	11.63	10.65	1	1	Ncam1	neural cell adhesion molecule 1
17383052	7.32	8.32	7.29	1	1	Notch1	notch 1
17400813	9.04	9.04	9.85	1	1	Notch2	notch 2
17343299	6.19	8.62	6.83	1	1	Notch3	notch 3
17336559	5.95	6.1	6.1	1	1	Notch4	notch 4
17224684	5.99	5.34	6.19	1	1	Pax3	paired box 3
17431910	8.26	8.83	7.39	1	1	Pax7	paired box 7
17419277	9.94	10.29	9.74	1	1	Sdc3	syndecan 3
17394175	9.84	8.95	9.06	1	1	Sdc4	syndecan 4
17409649	8.7	6.77	7.36	1	1	Vcam1	vascular cell adhesion molecule 1

Table S1: Microarray Analysis of MuSC-featured Genes in Culture activated Satellite Cells (Cult-ASC), Fibrin-expanded Satellite Cells (FSC-d7), and Fibrin-expanded bulk Skeletal Muscle Cells (FBC), Related to Figure 2

Table S2: Primer Sequences

Primers	***sequences (5'-3')***
Primers for PCR	
Pax7-F	GCTTGCCCACGTCCCAGTCTT
Pax7-R	ACAGCGGAGTGTTCCCCAAGC
Integrin צ$_{V}$-F	GCCTATTGTTCAGCACATATATGAGCTGAG
Integrin צ$_{V}$-R	CCAGGTGATGTTAGTGGTGACTAGTGTAG
Integrin ק$_{3}$-F	AGATGTGTTCCGGCCATGGGC
Integrin ק$_{3}$-R	ACAGCGGGTTGTTTGCTGTGTC
HPRT-F	CTCATGGACTGATTATGGACAGGAC
HPRT-R	GCAGGTCAGCAAAGAACTTATAGCC
